# Robotic thoracic surgery training in the UK and Republic of Ireland: national survey of trainee exposure, preparedness, and barriers

**DOI:** 10.1007/s11701-026-03511-5

**Published:** 2026-05-27

**Authors:** Hannah S. Jesani, Aaron S. Hundle, Michael R. Gooseman, Akshay J. Patel, SoRTS Collaborators

**Affiliations:** 1 Department of Cardiothoracic Surgery, University Hospital Coventry and Warwickshire NHS Trust, Coventry, UK; 2 Department of Medical Education, University Hospitals Birmingham NHS Foundation Trust, Birmingham, UK; 3 Department of Thoracic Surgery, Hull University Teaching Hospitals NHS Trust, Hull, England UK; 4 Department of Thoracic Surgery, Guy’s Hospital, Guy’s and St. Thomas’ NHS Foundation Trust, London, UK; 5 Institute of Immunology and Immunotherapy, University of Birmingham, Birmingham, UK

**Keywords:** Robotic-assisted thoracic surgery, Robotic surgery, Cardiothoracic surgery, Surgical training, Simulation, Surgical education

## Abstract

Robotic-assisted thoracic surgery (RATS) is increasingly being incorporated into contemporary practice across the United Kingdom (UK) and Republic of Ireland (ROI). However, national data on trainee exposure and preparedness is lacking. This study is the first to evaluate cardiothoracic trainees’ experiences and perceptions of training for RATS nationally. A multicentre, cross-sectional survey study was conducted between June and October 2025 of cardiothoracic trainees (ST1–ST8), post-certificate fellows, and equivalent trust appointed doctors. A 30-item, web-based questionnaire assessed institutional exposure, simulation access, operative console experience, and perceived barriers to RATS training. Quantitative data were analysed using descriptive statistics; while free-text responses underwent inductive thematic analysis. Eighty-two responses were received from all 14 training deaneries. RATS was performed in 71% (58/82) of respondents’ units; however only 16% (9/58) reported a structured training programme in place. Dual console systems were absent or unused in 43% (25/58) of robotic units. Over half (54%, 32/59) of respondents had not fully performed a single RATS procedure. Most respondents (83%, 68/82) agreed that more robotic training is required before the completion of training. Key barriers to training included lack of dual-console utilisation, operative exposure limited to bedside assisting only, and restricted simulation access. Despite widespread adoption of RATS, opportunities for structured training remain inconsistent across the UK and ROI. National standards should be developed to incorporate structured robotic training within the cardiothoracic curriculum, including access to simulation, routine dual-console use, and competency-based progression from bedside assistant to primary operator.

**Supplementary Information:**

The online version contains supplementary material available at 10.1007/s11701-026-03511-5.

## Introduction

Robotic-assisted thoracic surgery (RATS) has transitioned from an emerging technology to an integral part of contemporary practice. Across the United Kingdom (UK) and Republic of Ireland (ROI), robotic platforms are now established in an increasing number of thoracic units, with broadening indications and growing case volumes. This mirrors international trends and reflects recognised advantages of robotic surgery, which include enhanced visualisation, improved instrument articulation, and favourable ergonomics for the operating surgeon [1–4]. The adoption of innovative surgical techniques in thoracic surgery poses distinct challenges. Surgeons must now develop advanced psychomotor skills with a novel operative interface, applying different cognitive frameworks from conventional open or video-assisted thoracoscopic surgery. Without a structured, longitudinal training pathway, resident doctors may emerge from training with insufficient preparation for consultant-level practice in this field.

In England, the Getting It Right First Time report in 2025 on robotic-assisted surgery identified variability in access to robotic training and recognised training is often uncoordinated and misaligned to trainee needs [5]. Recommendations highlighted training should be embedded within robotic service development rather than retrospectively implemented, with emphasis on education through dual-console systems, high-fidelity simulation, structured programmes, and regional bootcamps [5].

Focusing on RATS, international surveys from the United States and Europe have revealed marked heterogeneity in trainee exposure, limited hands-on console time, and reliance on opportunistic rather than curriculum-driven learning [6–8].These studies advocate for formalised training programmes incorporating simulation, staged console progression, and objective assessment of competency [6–8]. No equivalent national evaluation of experience in RATS training has been undertaken within the UK or ROI. Therefore, the extent of trainee exposure to RATS, access to structured training, and perceived preparedness for independent robotic practice are currently unknown. The primary aim of this multicentre survey study was to evaluate the current experiences and perceptions of training in RATS among cardiothoracic trainees across the UK and ROI.

## Methods

### Study design

This study was a multicentre, cross-sectional, web-based survey administered using Microsoft Forms (Microsoft Corporation, Redmond, WA, USA). We have followed the Consensus-Based Checklist of Reporting of Survey Studies (Table S2) [9]. Data collection was conducted over a pre-defined period from 27 June 2025 to 27 October 2025.

### Participants

Eligible participants were any resident doctor who had worked in a cardiothoracic surgery department within the UK or ROI between August 2024 and August 2025. This included national cardiothoracic trainees (ST1-ST8), fellows after Certificate of Completion of Training (post-CCT), and trust appointed doctors at equivalent grades. Exclusion criteria were foundation doctors or consultants.

### Survey development and dissemination

The survey included a total of 30 question items (Table S3) designed to capture quantitative and qualitative data relating to trainees’ perspectives of training in RATS. The survey was divided into three domains including respondent demographics; experience of robotic training; and operative exposure in RATS. The question items were reviewed by consultant supervisors from the project steering committee and refined after trial with a small pilot cohort of eligible participants. The education group from national professional body the Society for Cardiothoracic Surgery in Great Britain and Ireland (SCTS) circulated the survey through established communication channels. Additionally, a network of regional collaborators referred to as the Survey of Robotic Thoracic Surgery (SoRTS) UK Collaborative Network, were recruited from training deaneries to promote the survey using standardised adverts.

### Data analysis

Survey completion required a response input to all questions, except for those relating to demographic characteristics, thereby preventing missing data entry. Quantitative data were analysed using Microsoft Excel (Version 16.107, Microsoft Corporation) and reported as frequencies, percentages, and measures of central tendency or dispersion as appropriate. Qualitative data were analysed using NVivo (Version 14, Lumivero) following an inductive thematic analysis approach to categorise themes and codes based on consensus within free-text responses [10].

### Ethical considerations

Approval was granted for conducting this survey-based evaluation of training practice according to governance protocols at Hull University Teaching Hospitals NHS Trust. Only the project steering committee members had access to original survey responses, which were then pseudo-anonymised by allocation of a unique respondent identifier (RID). No identifiable data is reported in the final manuscript.

## Results

### Participant demographics

**Fig. 1:**
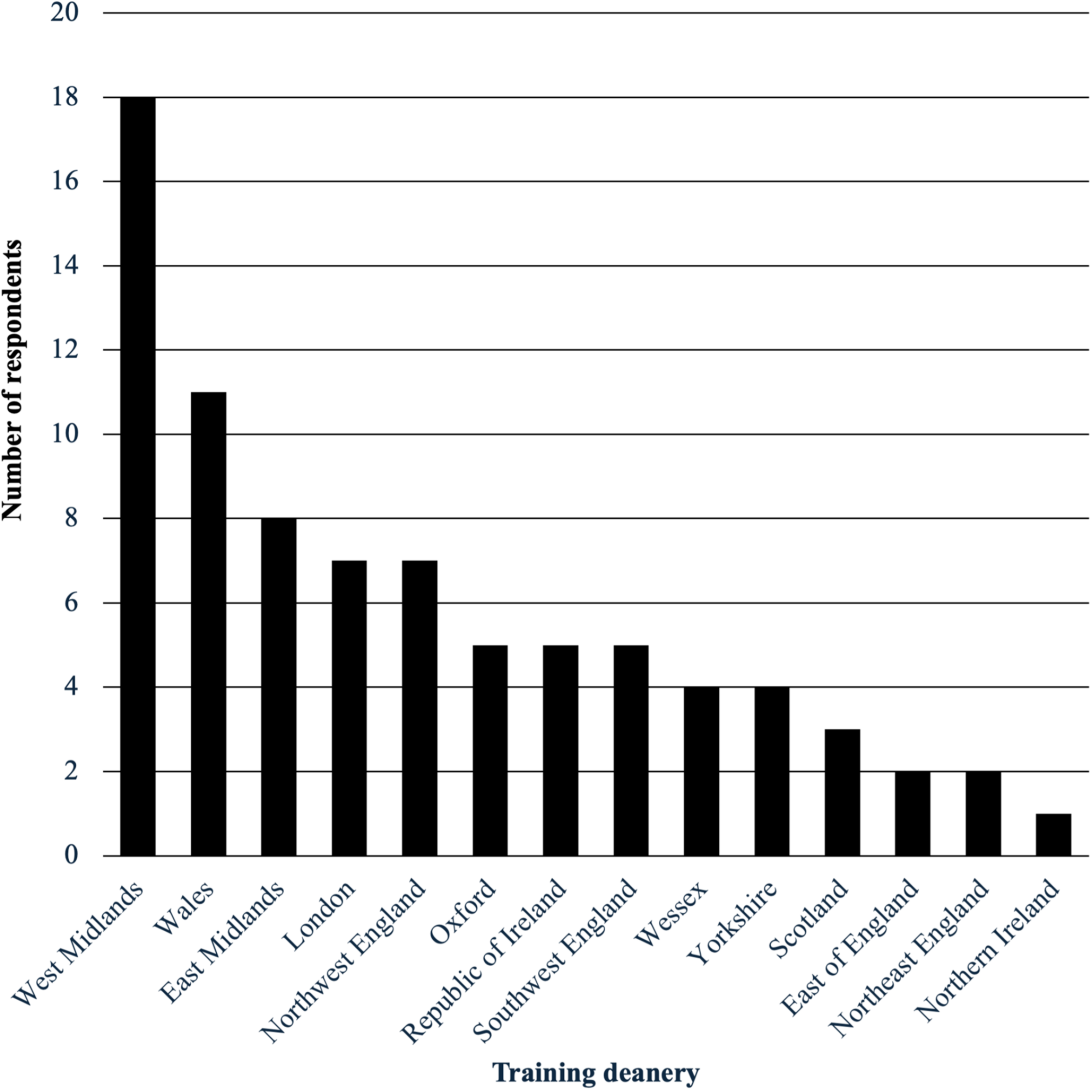
Bar chart showing number of eligible survey responses from all different training regions in the United Kingdom and Republic of Ireland

The survey received a total of 82 eligible responses, with representation from all 14 training deaneries nationally (Fig. 1). There was one exclusion due to a duplicate entry. Participants included national cardiothoracic trainees at ST1-ST8 level (61%, 50/82), non-trainees at equivalent grades (37%, 30/82) and post-CCT fellows (2%, 2/82). Gender distribution showed that 70% (56/80) identified as male, with median age 34 years (range 25–69). Participants most commonly graduated from the UK for their primary medication qualification (46%, 36/78), followed by Egypt (17%, 13/78) and ROI (8%, 6/78). The remaining 29% (23/78) were international medical graduates from a wide range of countries globally. Enquiry into career intentions demonstrated that 66% (54/82) had chosen thoracic surgery as their subspecialty. Of these respondents 94% (51/54) indicated they would like to incorporate robotic surgery into their consultant practice. Overall, 62% (51/82) of respondents were interested in applying for, or had already undertaken, a fellowship in RATS.

### Experience of robotic training

A total of 71% (58/82) of respondents reported that RATS was being performed at their department. Among these, 69% (40/58) had received formal training in bedside assisting for robotic cases, yet only 16% (9/58) reported that their department had provided a structured training programme for performing robotic surgery. There were a total of 31 separate thoracic surgery units represented in this survey. Among these 23% (7/31) centres were not performing RATS at the time of this survey. The most commonly used robotic surgical system was Da Vinci (Intuitive Surgical) (95%, 54/58), with a smaller proportion using Versius (CMR Surgical) (3%, 2/58), and just one respondent declared the use of both robotic systems. 43% (25/58) of respondents revealed that they either did not have a dual-console arrangement, or it was not used despite being available at their department (Table 1). Overall, respondents reported a median of 5 h (interquartile range, IQR 0–20, range 0–80) of simulation training on a robotic console, whilst 48% (39/82) had attended a formal course in robotic surgery. A majority of respondents (83%, 68/82) were in favour that more thoracic surgery training should be provided before the completion of training.

**Table 1:** Utilisation of console system operating arrangements (total respondent *n* = 58)

Console arrangement reported by respondents	Number
Not sure of arrangement	9 (16%)
Not applicable or only single console arrangement	15 (26%)
Dual console arrangement never used despite being available	10 (17%)
Dual console arrangement used once monthly	6 (10%)
Dual console arrangement used once weekly	11 (19%)
Dual console arrangement used for every case	7 (12%)

### Operative exposure in RATS

The majority of respondents (72%, 59/82) had experienced thoracic surgery as their main operating subspeciality between the timeframe of August 2024 and August 2025. Among these, over half the respondents (54%, 32/59) were provided no training opportunity to fully perform robotic procedures directly. The role of bedside first assistant during robotic procedures was most commonly occupied by the specialty registrar or senior resident doctors, followed by surgical care practitioners, then junior resident doctors. Respondents reported being bedside assistant for a median of 20 robotic cases (IQR 0–30, range 0-100), partly performing a median of 0 robotic cases (IQR 0–5, range 0–35), and fully performing a median of 0 robotic cases (IQR 0–5, range 0–80). Of those who had been able to perform robotic procedures, they were most commonly for minor cases such as wedge resection or bullectomy.

### Perspectives on the structure of training

**Fig. 2:**
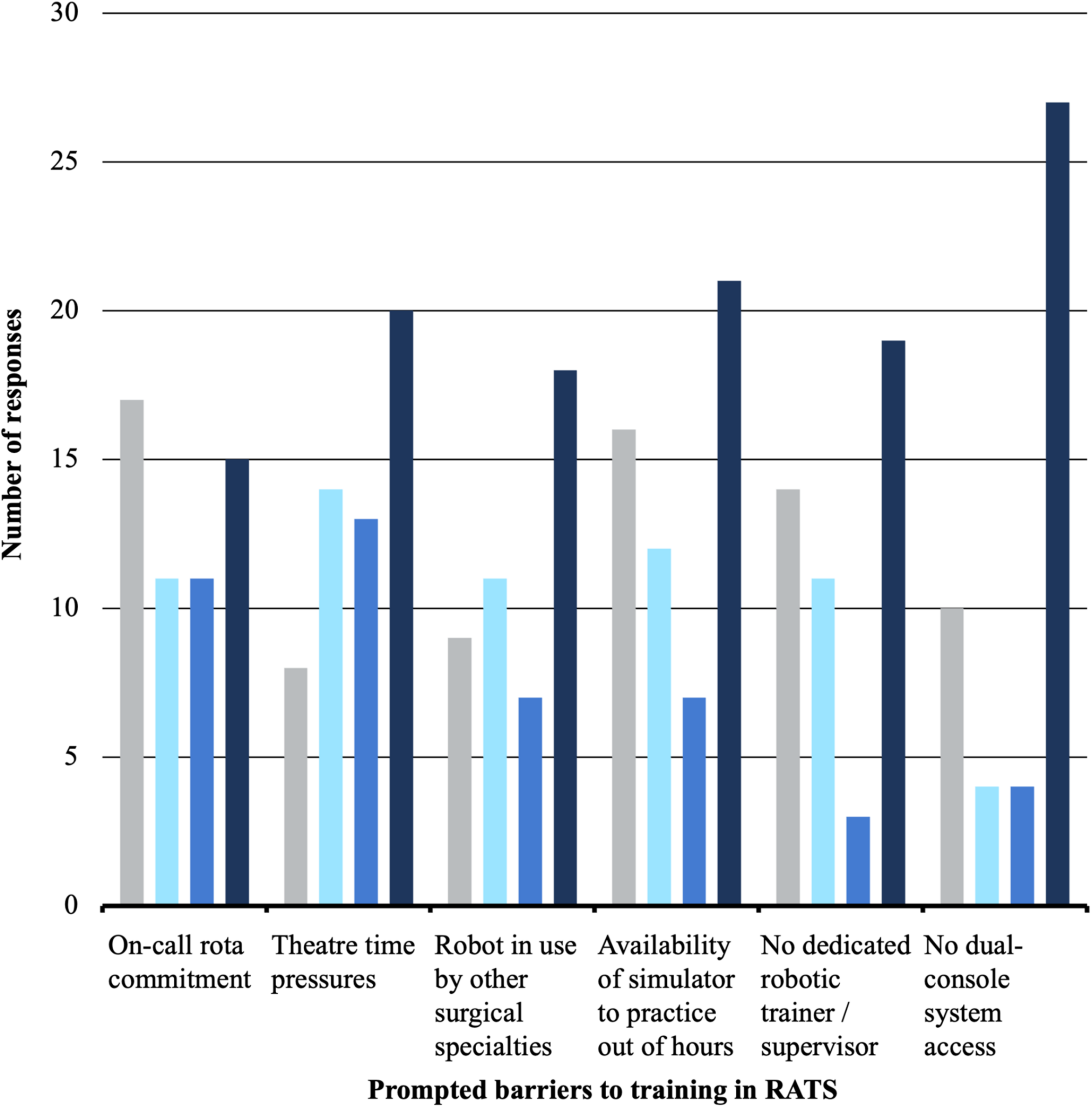
Respondents’ rating of significance level for perceived barriers to training in RATS. Bar chart excludes ‘not applicable’ response choices (total respondent *n* = 82)

Question item 24 explored the relevance of a series of prompted barriers to training in RATS. Respondents identified a lack of dual-console system access as the most significant barrier (Fig. 2). Other barriers identified as significant were time constraints in theatre and restricted access to robotic consoles for simulation sessions.

Thematic analysis of question items 23 and 25 identified 11 codes within the theme of ‘*what respondents found valuable about their current training’* (Table 2), and 11 codes within the theme of *‘how respondents would like their training to be improved’* (Table 3). There were clear parallels in codes across these two opposing themes, which reflected respondents’ experiences at units that were delivering beneficial features of a structured training programme versus those that were not. Many of these codes aligned with the six prompted barriers from question item 24. Key additional codes that emerged from respondents’ perspectives on how to improve training included providing a structured programme, prioritising other members of the multidisciplinary team for bedside assistant role allocation, and offering earlier exposure to RATS through opportunities such as courses and fellowships, with industry support.

**Table 2:** Frequency breakdown of codes, with supporting quotes, within the theme of ‘what respondents found valuable about their current training’ in RATS. RID: respondent identifier

Theme: ‘what respondents found valuable about their current training’ in RATS		
Code	Number of references	Supporting quotes from responses to survey question items
Innovation exposure	11	‘This experience has strengthened my interest in minimally invasive cardiothoracic surgery and has motivated me to pursue further training in this field as it clearly represents the future of thoracic surgery.’ (RID: 25) ‘Robotic surgery is the present and future. I find it valuable to receive any training which can help me be a future thoracic consultant equipped with the skills I need.’ (RID: 74)
Trainer support	8	‘We have an extremely good programme where I have essentially done a fellowship in training. The consultants are all very good trainers, one is also a proctor.’ (RID: 62)
Appreciation of surgical anatomy	6	‘…but more importantly in appreciating the anatomy and manipulation within the chest. It is unparalleled and significantly accelerates an individual’s conceptual understanding of thoracic anatomy.’ (RID: 11)
Dual-console system access	6	‘Dual console operation lends more training opportunities’ (RID: 40)
Early operative exposure	6	‘I think [RATS] is a very important tool that should be a priority for any trainee regardless [of] the level.’ (RID: 73)
Surgical precision and technical skill development	6	‘Additionally, I’ve seen how robotics challenges you to be meticulous and deliberate. Even assisting with port placement and docking taught me the importance of planning and spatial orientation.’ (RID: 25)
Structured training approach	6	‘I’ve also found it useful when I’ve had stepwise learning e.g. port placement, then initial lung retraction, lymph node dissection, then progressing to hilar structure dissection etc.’ (RID: 65)
Simulation	5	‘Simulation training is very useful’ (RID: 49)
Allocation of bedside assistant roles	4	‘Having SHO/SCP available to allow trainee to be on console.’ (RID: 8) ‘The SCPs bedside assist has been invaluable’ (RID: 28)
Operative ergonomics	4	‘…ergonomic benefits robotic systems offer, especially in complex resections like segmentectomies or mediastinal mass excisions.’ (RID: 25)
Courses	2	‘Excellent training days in Robotic thoracic surgery have been provided in the Royal College of Surgeons, Ireland with hands on learning’ (RID: 42)

**Table 3:** Frequency breakdown of codes, with supporting quotes, within the theme of ‘how respondents would like their training to be improved’ in RATS. RID: respondent identifier

Theme: ‘how respondents would like their training to be improved’ in RATS		
Code	Number of references	Supporting quotes from responses to survey question items
Dual-console system access	20	‘Dual consoles are excellent tools to training. I think they give both trainees and trainers the comfort and peace of mind to build confidence and drive experience. They should be available and used in all thoracic surgical centres.’ (RID: 63)
Structured training approach	18	‘A stepwise formal training pathway that provides trainees an opportunity to perform cases or part of cases on the console, once a certain amount of simulation and bedside assisting has been completed’ (RID: 42) ‘A dedicated structured training pathway with steps towards progression would be valuable with robotic surgeon proctor.’ (RID: 70)
Theatre time allocation	15	‘List should be modified to account for training cases (i.e. reduce the number of cases in a trainee list so that time pressure isn’t so significant).’ (RID: 71)
Trainer support	14	‘By increasing time available for registrars to be able to operate using robots under close supervision from experienced consultants without the added stress of time constraints.’ (RID: 79)
Allocation of bedside assistant roles	11	‘Additionally, having a Surgical Care Practitioner (SCP) on site to assist would enhance efficiency and support the operating team. At present, one registrar acts in an assisting role while another, often closer to the consultant, takes the lead for training or promotion. Reviewing and balancing such arrangements could help ensure fairer distribution of hands‑on experience and progression opportunities.’ (RID: 64) ‘…lack of bedside assistants so registrar trainees have less opportunity to undertake cases with direct consultant supervision and training’ (RID: 70)
Simulation	10	‘…protected time for simulation hours’ (RID: 46) ‘structured simulation sessions integrated into training’ (RID: 67)
Courses	6	‘Dedicated training courses and sessions should be available in the hospital’ (RID: 58)
Fellowships	3	‘More robotic fellowships’ (RID: 65)
Early operative exposure	2	‘It should be added earlier in the training program with proper set goals’ (RID: 26)
Support for non-trainees	2	‘A tiered training model could work well - where NTNs receive priority for full console training, but dedicated non-training fellows are given scheduled opportunities to participate meaningfully, especially when they’ve demonstrated long-term interest and commitment to the specialty.’ (RID: 25)
Industry support	1	‘more involvement from the industry to help facilitate this’ (RID: 15)

## Discussion

This national survey represents the first comprehensive evaluation of cardiothoracic trainees’ experiences in robotic thoracic surgery training across the UK and ROI. As robotic platforms become increasingly embedded within thoracic surgical practice, our findings demonstrate a clear mismatch between the pace of technological adoption and the availability of structured training opportunities.

### Structured training approach

Respondents expressed a desire for more robotic training prior to consultant practice, with a dedicated, stepwise training pathway among the most called for improvements (Table 3). In 2018, a Delphi consensus statement by the European Society of Thoracic Surgeons (ESTS) and European Association of Cardiothoracic Surgery (EACTS) set out clear recommendations for robotic thoracic training [11]. The robust framework defined training phases aligned to a recognised learning curve, advocated for mandatory dry-lab and simulation-based training, and advised supervised console exposure with formal proctored assessment prior to independent practice [11]. Nevertheless, this was mainly focused on consultant-level training rather than for resident doctors. This is reflected in our results demonstrating respondents’ limited exposure to a structured training approach. Only a small proportion (21%, 12/58) reported access to an embedded structured RATS training programme, or such a programme being in development at their training unit. A 2025 consensus by the Society of Thoracic Surgeons (STS) further emphasised a structured, longitudinal approach outlining 12 core components including simulation, competency assessment and credentialing [6, 7]. The central recommendation of a structured training approach is echoed by European publications [8, 12, 13], and highlights the need for similar adoption in the UK and ROI.

### Dual-console system access

The routine use of dual-console systems for teaching cases is a key component shared across published consensus frameworks [7, 11–13]. Our analysis identified discordance, with limited access to dual-console systems as a major barrier to training experiences (Table 3). We acknowledge that not all robotic surgical systems offer a dual-console arrangement, however even amongst units where the dual-console systems were available, trainees reported inconsistent utilisation during cases (Table 1). Capital investment alone is insufficient without parallel changes in operating culture. Although the implementation of dual-console robotic systems are dependent on institutional priorities and local resources allocation, thoracic surgery service design should adopt the use of dual-console systems in all thoracic units undertaking robotic surgery. Despite the dominance of the DaVinci robotic system used nationally, it is appropriate for structured training programmes to be system-agnostic to ensure equity. The high initial financial burden of investment in robotic systems is potentially justified through increased productivity [4]. Theatre time pressures can impact on training needs and there is increased scrutiny on efficiency in the current clinical climate. It is therefore essential the robotic theatre environment is optimised to provide capacity to support training, whilst maintaining patient safety and proficiency.

### Allocation of the bedside assistant role

Another key STS recommendation is the provision of a dedicated, trained bedside assistant during training cases [7]. However, our survey has demonstrated that senior resident doctors are frequently allocated to the bedside assistant role (72%), often limiting operating experience. Many respondents noted that the lack of a separate, trained robotic bedside assistant had impacted their training (Table 3). Integration of dedicated surgical care practitioners (SCPs) into robotic programmes can provide consistent bedside expertise, improve operating theatre efficiency, and facilitate trainee’s transition from bedside assistant to console surgeon [14]. Theatre team efficiency is key to improving theatre flow and allowing adequate time for training needs particularly familiarity and efficiency of the theatre team in setting up the robotic system. However, not all respondents had received formal bedside assistant training themselves (69%, 40/58). This demonstrates national variation in preparation for a role that is technically demanding and yet critical to safe, effective robotic surgery.

### Simulation training

Technical skills simulation is an important preparatory and adjunctive step to operative learning in vivo. Despite this, a lack of respondents’ regular access to robotic consoles for simulation is shown by the median amount of simulation performed at just 5 h (IQR 0–20, range 0–80). Training rotas could be designed with consistent, protected time to access simulators for skill progression. Non-technical skills for surgeons (NOTSS) are also fundamental for safe robotic practice [15]. They are highly relevant to the complex task of RATS in which good command of decision-making, communication and situational awareness is required, especially when converting to an open technique in the management of challenging cases with dense adhesions or major bleeding. A European survey identified teamwork and communication as the most critical non-technical domains in RATS, yet highlighted a lack of structured, team-based training in place [12, 16]. This aligns with STS recommendations for incorporation of formal NOTSS training, including simulation-based exercises for the robotic theatre team [6, 7].

### Timing of exposure to RATS

The high proportion of respondents indicating an intention to pursue robotic thoracic fellowships (62%, 51/82) suggests perceived deficits in the current standard of robotic training pathways. Implementation of a robotic thoracic fellowship programme has been shown to facilitate safe and effective training [17]. However, reliance on post-CCT fellowships risks entrenching inequity in access to robotic expertise. Fellowship training is intended to consolidate and extend specialist competencies; it should not be relied upon to address gaps in specialty training but allow greater technical exposure and skills to enhance trainees’ skills [18]. Meanwhile, the paucity of RATS primary operator exposure in current training pathways is evident from the low average number of robotic cases that were partly or fully performed by respondents. The data showed a skewed distribution indicating that only a minority of respondents gained substantial operative exposure. Overall, trainees identified that earlier exposure to RATS was a valued and desirable feature during training (Tables 2 and 3), which would in turn increase the value from undertaking a subsequent robotic fellowship. Another factor contributing to variability in trainee exposure is that consultant trainers are often progressing through different stages of their learning curve in performing RATS [19]. We would expect consultants to become more willing and able to train resident doctors as their own experience develops.

### Future training in robotic-assisted surgery

The report of the Future of Surgery Technology Enhanced Surgical Training Commission signals a paradigm shift in surgical training, focusing attention on how technological innovation can enhance training and aid the surgical workforce [20]. The Royal College of Surgeons of England has explicitly reported their priorities for training the next generation of robotic surgeons, recognising its potential for improving operative practices, but also varying level of adoption across different surgical specialties [21]. The Royal College of Surgeons of Ireland have reached a significant milestone in standardising robotic training by launching their first National Robotic Surgery Curriculum [22]. Specialties such as urology and general surgery have paved the way for establishing competency-based curricula and structured assessment tools for robotic surgery in their national training pathways [23–26].

We anticipate an exciting period ahead of progression in thoracic surgery training, with substantial opportunities for innovation, collaboration and educational refinement [20, 21]. A future aim is to utilise insights from our national study to support the development of national guidelines for robotic surgery training that can be integrated into the cardiothoracic higher specialty curriculum. There have already been some steps towards formally integrating robotic surgery competencies into the Intercollegiate Surgical Curriculum Programme, including the addition of robotic-specific components to procedural-based assessments [27]. This enables structured skills logging, such as robot docking, console time, and graded participation in robotic procedures. Overall, our findings reinforce that RATS training programmes implemented in the UK and ROI should incorporate access to dual-console systems, protected simulation-based training and dedicated trainer supervision for competency-based assessments.

### Limitations

A target population size inferred from the 2025 SCTS cardiothoracic surgery workforce report is 141 nationally appointed resident doctors [28], response rate amongst these trainees was approximately 35%. The survey methodology was at risk of selection and response bias, with the possibility that trainees with a particular interest in or exposure to RATS were more likely to participate. Addressing this, no exclusions were applied to respondents based on their subspecialty career intention or prior robotic experience to capture a more representative and broad range of perceptions from the target population. Recruiting respondents through the SoRTS UK Collaborative Network also improved the comprehensive reach of the survey nationally. However, response rate varied significantly between training regions which may limit the generalisability of findings. We did not perform a subgroup analysis on geographical access to robotic training. However, the Royal College of surgeons of England has recently recognised the “postcode lottery” in terms of patient’s access to robotic-assisted surgery in the NHS [29]. We have observed a comparable national disparity in access to robotic thoracic surgery from a training perspective, which aligns with recommendations in the college’s statement for a clearer, more consistent funding model and a continued focus on training [29]. Since data is self-reported at a single time point, it represents respondents’ perceived experience and may not fully reflect the rapidly evolving landscape of RATS training. Undertaking the survey again at future time periods to reassess the state of training may be valuable as practice patterns change. Despite these limitations, this survey is the first to explore cardiothoracic trainees’ perspectives in the UK and ROI, providing important national insights into current experience and desired training needs for RATS.

## Conclusion

This national multicentre survey highlights enthusiasm among cardiothoracic resident doctors for greater exposure to RATS as well as the provision of structured training programmes. We have provided the first evaluation in the UK and ROI of trainee access to RATS procedures, opportunities for simulation, facilitators of effective training and perceived barriers in progression towards safe, independent robotic practice. Important barriers that were identified by respondents included limited access to dual-console systems, allocation to bedside assistant roles only, and reduced availability of robotic consoles for simulation-based practice. These findings suggest that increased integration of robotic-assisted surgery into thoracic practice has not yet been matched by consistent training opportunities for resident doctors. Professional bodies have already declared their commitment to structured robotic training, meanwhile our findings offer focus for meaningful reform of training pathways. The barriers we have identified can be addressed through cultural change, strategic investment in infrastructure, protected training time and dedicated robotic training supervisors to ensure equitable, competency-based robotic training. Further collaborative work between training bodies, professional societies and robotic centres can inform the development of a national framework. Such a framework would provide a foundation to standardise training, reduce regional disparities and ensure that the next generation of cardiothoracic consultants are equipped to deliver safe, high-quality robotic surgical practice.

## Electronic Supplementary Material

Below is the link to the electronic supplementary material.

Supplementary Material 1

## Data Availability

No datasets were generated or analysed during the current study.

## Declarations

Approval was granted for conducting this survey-based evaluation of training practice according to governance protocols at Hull University Teaching Hospitals NHS Trust.
