# Supplementary material for: Robotic thoracic surgery training in the UK and Republic of Ireland: national survey of trainee exposure, preparedness, and barriers
Source: J Robot Surg. 2026 May 27;20(1):543. doi: 10.1007/s11701-026-03511-5 (PMC13216105; doi:10.1007/s11701-026-03511-5)
Supplement: Supplementary file 1 — Supplementary Material 1 [file 11701_2026_3511_MOESM1_ESM.docx]

**Supplementary Material**

**Table S1:** Survey of Robotic Thoracic Surgery (SoRTS) Collaborators

| **Name** | **Representing Deanery** |
| --- | --- |
| Karishma Chandarana | East Midlands |
| Nicole Asemota | East of England |
| Hanan Hemead | Northwest England |
| Ali Ansaripour | Oxford |
| Jack Whooley | Republic of Ireland |
| Sanjeet Singh | Scotland |
| Saima Azam | Scotland |
| Rhian Allen | Wales |
| Ahmed El Zeki | Wessex |
| Moslem Abdelghafar | Wessex |

**Table S2:** Checklist for Reporting Of Survey Studies (CROSS) Reporting Guideline

Sharma A, Minh Duc NT, Luu Lam Thang T, Nam NH, Ng SJ, Abbas KS, et al. A Consensus-Based Checklist for Reporting of Survey Studies (CROSS). Journal of General Internal Medicine. 2021 Apr 22;36(10):3179–87.

| **Section/topic** | **Item** | **Item description** | **Reported on page #** |
| --- | --- | --- | --- |
| **Title and abstract** | | |  |
| **Title and abstract** | **1a** | **State the word “survey” along with a commonly used term in title or abstract to introduce the study’s design.** | 1 |
|  | **1b** | **Provide an informative summary in the abstract, covering background, objectives, methods, findings/results, interpretation/discussion, and conclusions.** | 3 |
| **Introduction** | | |  |
| **Background** | **2** | **Provide a background about the rationale of study, what has been previously done, and why this survey is needed.** | 5-6 |
| **Purpose/aim** | **3** | **Identify specific purposes, aims, goals, or objectives of the study.** | 6 |
| **Methods** | | |  |
| **Study design** | **4** | **Specify the study design in the methods section with a commonly used term (e.g., cross-sectional or longitudinal).** | 6 |
|  | **5a** | **Describe the questionnaire (e.g., number of sections, number of questions, number and names of instruments used).** | 6-7 |
| **Data collection methods** | **5b** | **Describe all questionnaire instruments that were used in the survey to measure particular concepts. Report target population, reported validity and reliability information, scoring/classification procedure, and reference links (if any).** | 6-7 |
|  | **5c** | **Provide information on pretesting of the questionnaire, if performed (in the article or in an online supplement). Report the method of pretesting, number of times questionnaire was pre-tested, number and demographics of participants used for pretesting, and the level of similarity of demographics between pre-testing participants and sample population.** | 6-7 |
|  | **5d** | **Questionnaire if possible, should be fully provided (in the article, or as appendices or as an online supplement).** | Table S3 |
| **Sample characteristics** | **6a** | **Describe the study population (i.e., background, locations, eligibility criteria for participant inclusion in survey, exclusion criteria).** | 6-8 |
|  | **6b** | **Describe the sampling techniques used (e.g., single stage or multistage sampling, simple random sampling, stratified sampling, cluster sampling, convenience sampling). Specify the locations of sample participants whenever clustered sampling was applied.** | 6-7 |
|  | **6c** | **Provide information on sample size, along with details of sample size calculation.** | 6, 20 |
|  | **6d** | **Describe how representative the sample is of the study population (or target population if possible), particularly for population-based surveys.** | 6, 20 |
| **Survey**  **administration** | **7a** | **Provide information on modes of questionnaire administration, including the type and number of contacts, the location where the survey was conducted (e.g., outpatient room or by use of online tools, such as SurveyMonkey).** | 6 |
|  | **7b** | **Provide information of survey’s time frame, such as periods of recruitment, exposure, and follow-up days.** | 6 |
|  | **7c** | **Provide information on the entry process:**  **–>For non-web-based surveys, provide approaches to minimize human error in data entry.**  **–>For web-based surveys, provide approaches to prevent “multiple participation” of participants.** | 6-7 |
| **Study preparation** | **8** | **Describe any preparation process before conducting the survey (e.g., interviewers’ training process, advertising the survey).** | 6 |
| **Ethical considerations** | **9a** | **Provide information on ethical approval for the survey if obtained, including informed consent, institutional review board [IRB] approval, Helsinki declaration, and good clinical practice [GCP] declaration (as appropriate).** | 7 |
|  | **9b** | **Provide information about survey anonymity and confidentiality and describe what mechanisms were used to protect unauthorized access.** | 7 |
| **Statistical**  **analysis** | **10a** | **Describe statistical methods and analytical approach. Report the statistical software that was used for data analysis.** | 7 |
|  | **10b** | **Report any modification of variables used in the analysis, along with reference (if available).** | n/a |
|  | **10c** | **Report details about how missing data was handled. Include rate of missing items, missing data mechanism (i.e., missing completely at random [MCAR], missing at random [MAR] or missing not at random [MNAR]) and methods used to deal with missing data (e.g., multiple imputation).** | 7 |
|  | **10d** | **State how non-response error was addressed.** | n/a |
|  | **10e** | **For longitudinal surveys, state how loss to follow-up was addressed.** | n/a |
|  | **10f** | **Indicate whether any methods such as weighting of items or propensity scores have been used to adjust for non-representativeness of the sample.** | n/a |
|  | **10g** | **Describe any sensitivity analysis conducted.** | n/a |
| **Results** | | |  |
| **Respondent characteristics** | **11a** | **Report numbers of individuals at each stage of the study. Consider using a flow diagram, if possible.** | 8 |
|  | **11b** | **Provide reasons for non-participation at each stage, if possible.** | n/a |
|  | **11c** | **Report response rate, present the definition of response rate or the formula used to calculate response rate.** | 8, 20 |
|  | **11d** | **Provide information to define how unique visitors are determined. Report number of unique visitors along with relevant proportions (e.g., view proportion, participation proportion, completion proportion).** | n/a |
| **Descriptive**  **results** | **12** | **Provide characteristics of study participants, as well as information on potential confounders and assessed outcomes.** | 8 |
| **Main findings** | **13a** | **Give unadjusted estimates and, if applicable, confounder-adjusted estimates along with 95% confidence intervals and p-values.** | n/a |
|  | **13b** | **For multivariable analysis, provide information on the model building process, model fit statistics, and model assumptions (as appropriate).** | n/a |
|  | **13c** | **Provide details about any sensitivity analysis performed. If there are considerable amount of missing data, report sensitivity analyses comparing the results of complete cases with that of the imputed dataset (if possible).** | n/a |
| **Discussion** | | |  |
| **Limitations** | **14** | **Discuss the limitations of the study, considering sources of potential biases and imprecisions, such as non-representativeness of sample, study design, important uncontrolled confounders.** | 20 |
| **Interpretations** | **15** | **Give a cautious overall interpretation of results, based on potential biases and imprecisions and suggest areas for future research.** | 15, 19-20 |
| **Generalizability** | **16** | **Discuss the external validity of the results.** | 20 |
| **Other sections** | | |  |
| **Role of funding source** | **17** | **State whether any funding organization has had any roles in the survey’s design, implementation, and analysis.** | 1 |
| **Conflict of interest** | **18** | **Declare any potential conflict of interest.** | 1 |
| **Acknowledgements** | **19** | **Provide names of organizations/persons that are acknowledged along with their contribution to the research.** | 21, Table S1 |

**Table S3:** Survey question items with corresponding response options

| **Number** | **Question Item** | **Data entry type** | **Response options and/or restrictions** |
| --- | --- | --- | --- |
| ***Demographics*** | | | |
| 1. | **Please provide a name as you would like it to appear on certificate of participation** | Free text | Name  Or prefer not to say |
| *2.* | **Please provide your email** | Free text | Email  Or prefer not to say |
| *3.* | **Which deanery are you based?** | Multiple choice, single selection | Each UK and ROI deanery listed as options |
| *4.* | **What hospital have you worked in from August 2024-August 2025?** | Free Text | 1. Hospital 2. Prefer not to say |
| *5.* | **What is your level of training at the time of the survey?** | Multiple choice, single selection | 1. ST1 2. ST2 3. ST3 4. ST4 5. ST5 6. ST6 7. ST7 8. ST8 9. Post-CCT Fellow 10. Non-trainee – Senior Fellow 11. Non trainee Junior Fellow |
| *6.* | **For Trainees, how many prior years of thoracic clinical experience did you have before entering training?** | Free text | Numerical only |
| *7.* | **For Non-Trainees, how many years of thoracic clinical experience do you have in total?** | Free text | Numerical only |
| *8.* | **Which subspecialty is your intent?** | Multiple choice | 1. Thoracic surgery 2. Cardiac surgery 3. Congenital surgery 4. Undecided |
| *9.* | **Are you interested in applying for a robotic thoracic surgery fellowship/have completed?** | Multiple choice | 1. Yes – interested to apply 2. Yes – have completed 3. No 4. Undecided |
| *10.* | **Do you intend to incorporate robotic thoracic surgery into your consultant career?** | Multiple choice | 1. Yes 2. No 3. Undecided |
| *11.* | **What is your gender identity?** | Multiple choice | Male  Female  Non-binary  Prefer not to say |
| *12.* | **What is your age (in years) at the time of the survey?** | Multiple Choice | Numerical only  Or prefer not to say |
| *13.* | **Where was your primary medical degree qualification?** | Multiple choice | United Kingdom  Republic of Ireland  Prefer not to say  Other (free text) |
| ***Training and Robotic Work*** | | | |
| *14.* | **Is robotic thoracic surgery being performed in your hospital?** | Multiple choice | 1. Yes 2. No 3. Not sure |
| *15.* | **Which robot consoles are available for thoracic surgery at your hospital?** |  | 1. CMR Versius 2. Intuitive Da Vinci 3. Medtronic Hugo RAS 4. Not sure 5. Not applicable – no robot |
| *16.* | **Is there a structured robotic training programme for trainees?** | Multiple choice | 1. Yes – structured training programme 2. No – robotic surgery available but no training 3. No – training available but not formally structured 4. Training programme In development 5. Not sure 6. Not applicable – no robot available |
| *17.* | **In your centre who is bedside first assistant during robotic thoracic procedures?** | Multiple choice | 1. Registrar/Senior clinical grade 2. SHO/Junior clinical grade 3. Cardiothoracic ACP/SCP 4. Consultant surgeon 5. Other 6. Not applicable – no robot available |
| *18.* | **Have you received formal training in bedside assisting for robotic cases? (e.g. first assist course)** | Multiple choice | 1. Yes 2. No |
| *19.* | **Have you attended any other formal robotic surgery courses? And if so what models were used?** | Multiple choice | 1. Yes – human cadaver 2. Yes- animal cadaver 3. Yes virtual simulation 4. No – I haven’t attended any other courses |
| *20.* | **How many hours of simulation training have you undergone on the robot console, outside of formal courses?** | Free text | Numerical only |
| *21.* | **How often is dual console arrangement used to support your training in robotic thoracic surgery?** | Multiple choice | 1. Not applicable – no robot available 2. Not applicable – only single console available 3. Never - despite dual console 4. At least once monthly 5. At least once weekly 6. For every robotic case 7. Not sure |
| *22.* | **To what extent do you agree with the following statement? “More thoracic robotic surgery training should be provided before fellowships (OPE) and CCT”** | Likert-scale, 5-point | 1. Strongly Agree 2. Agree 3. Neutral 4. Disagree 5. Strongly disagree |
| *23.* | **What do you find valuable about your current training in robotic thoracic surgery?** | Free Text | No restrictions |
| *24.* | **To what extent has each of the following been a significant barrier to your training in robotic thoracic surgery?**  •     Time constraints eg: rota on call commitments  •     Time constraints in theatre eg: pressure to finish on time  •     Availability of robot console eg: in use by other specialities  •     Availability of robot console eg: having to practice on simulator out of hours  •     Lack of support from trainers eg: no dedicated robotic supervisor  •     Lack of dual console operating | Likert scale 1-5 | 1. Not applicable 2. Slightly significant 3. Somewhat significant 4. Moderately significant 5. Highly significant |
| *25.* | **How would you like your training in robotic thoracic surgery to be improved?**  Welcome to state any other barriers | Free Text | No restrictions |
| ***Robotic experience*** | | | |
| *26.* | **What subspecialty have you mainly operated in for the past year (August 2024 – August 2025)** | Multiple choice | 1. Thoracic surgery 2. Cardiac surgery 3. Congenital surgery |
| *27.* | **How many robotic procedures have you been the bedside assistant for, in the last 12 months? (this includes duties such as docking, specimen retrieval, un-docking etc.)** | Free Text | Numerical only |
| *28.* | **How many robotic thoracic procedures have you partly performed (ST-S/ST-U), in the last 12 months? (on the console)** | Free text | Numerical only |
| *29.* | **How many robotic thoracic procedures have you fully performed (ST-S/ST-U/P), in the last 12 months?** | Free text | Numerical only |
| *30.* | **If Yes, which of these procedures have you performed operating on the console (ST-S, ST-US,P)?** | Multiple choice, select all that apply | 1. Not applicable 2. Minor (Wedge/Pneumothorax) 3. Mediastinal mass excision 4. Lung volume reduction surgery 5. Lobectomy 6. Segmentectomy |
